# SeqMonitor: Influenza Analysis Pipeline and Visualization

**DOI:** 10.1371/currents.RRN1040

**Published:** 2009-09-22

**Authors:** Norman MacDonald, Donovan Parks, Robert Beiko

**Affiliations:** ^*^Faculty of Computer Science, Dalhousie University and ^†^Dalhousie University

## Abstract

Unprecedented sequencing effort has led to daily submissions of influenza genomes to public repositories such as the NCBI GenBank. With the decreasing cost of genome sequencing, it is expected that rapidly evolving viruses such as influenza will be sampled in even greater depth in the future. Keeping analyses up to date and managing this data is a prime concern for researchers and public-health officials alike. We have developed an influenza sequence pipeline, polymorphism data warehouse, and an interactive web-based analysis program to assist in managing the flow of sequence data. The system provides a framework for studying polymorphic associations with various metadata, for downloading subsets based on metadata criteria, as well as for tracking polymorphisms geographically and temporally. SeqMonitor is accessible at http://ratite.cs.dal.ca/SeqMonitor.

## Introduction

 In early 2009, a triple-reassortant strain of the H1N1 serotype, here-in called S-OIV (also known as H1N1pdm), spread throughout the world, causing a pandemic. The first significant human outbreak of this strain occurred in La Gloria, Veracruz, Mexico in February 2009 [1] [2]. As of 6 Sep 2009, 3205 S-OIV-related deaths worldwide have been reported to the WHO [3]. Although treatment concerns have been prompted by resistance to the antiviral oseltamivir in the latest S-OIV strains, the virus largely remains sensitive to zanamivir [4]. Resistance to oseltamivir is often conferred by a single His274Tyr amino acid mutation in the neuraminidase gene, while reduced zanamivir sensitivity has recently been experimentally linked to a Gln136Lys mutation (N2 numbering). 

Systems recently developed and under development are allowing the quick identification of important, novel mutations using 3-d protein structures [5], as well as H3N2 antigenic-site-based vaccine prediction systems (http://influenza.nhri.org.tw/ATIVS/index.jsp)  [6].  This type of automated detection and monitoring of novel mutations affecting antigenicity, convergent evolution, and inter/intra-host reassortment needs to be performed on a continual basis on the ever-increasing dataset to keep abreast of new influenza threats. To this end, we have created an automated pipeline that can download the latest sequences from NCBI GenBank and add them to existing alignments of homologous sequences, as well as extract metadata such as antiviral resistance, collection date and location name. Each sequence can then be geotagged by an automated, user-verified extraction and querying engine which uses the GeoNames web service (http://www.geonames.org). The data are made available through our data warehouse and web application, SeqMonitor. The current version of SeqMonitor allows users to submit H1N1 protein sequences of the hemagglutinin or neuraminidase genes to a BLAST query, with the top matches being plotted on a geographic map. Novel and rare mutations of the query sequence can then be analyzed versus any subset of the data, defined for instance by oseltamivir resistance or country of collection. The geographic and related metadata files, along with the precomputed amino acid alignments constructed with the pipeline can be downloaded by users and processed by geographic and sequence data analysis packages such as GenGIS (http://kiwi.cs.dal.ca/GenGIS) [7] [8]. SeqMonitor can be accessed at http://ratite.cs.dal.ca/SeqMonitor.

## Methods 

### Data sources 

 All available data on the hemagglutinin and neuraminidase proteins of H1N1 human-host influenza from the NCBI Influenza Virus Resource are downloaded and provided as input to the pipeline. The GeoNames.org webservice was used to geotag records. Currently (12 September 2009), 3968 H1N1 hemagglutinin and 2889 neuraminidase records are available for analysis with Version 1.0 of SeqMonitor. 

 All of the code developed for this pipeline was written in Python, using the Biopython library version 1.5 [9].  The data warehouse is managed by MySQL version 5.1.29.  The web interface is implemented with the Django Python web framework version 1.0.  The system is composed of two main modules. The pipeline module parses and integrates sequence and location data from the NCBI GenBank and Geonames.org. The visualization and analysis module allows the results of the pipeline to be explored through the web and compared with user submitted sequences.

### Pipeline - Parser 

 In the parsing step, the pipeline extracts the location, date of collection, antiviral resistance tags, S-OIV outbreak inclusion, as well as each of the standard sequence identifiers (i.e. GI number, accessions). S-OIV outbreak inclusion is determined by the 2009 H1N1 Flu Outbreak NCBI project number, 37813. Due to the relatively unstructured format of the data, location, date of collection, and antiviral resistance information can be defined in different formats in various blocks. The EpiFlu or FluData block is checked first and is considered as the authority for each metadata field. If this block or some of the metadata cannot be found, then other fields in the record are searched, such as the source feature block, or within the free-text notes. As much information about the date of collection is extracted from the record as possible. If complete collection date is not available, then just the month or year may be extracted. If none of these fields is present within the record, year of collection is parsed from the strain identifier itself. 

 Often, antiviral resistance information is provided in a human-readable format in a free-text notes field. This field is automatically parsed by splitting the notes fields on commas and semi-colons, then looking for the terms “adamantane”, “oseltamivir” or “zanamivir”. If exactly one of those terms appears in a clause, then that clause is searched for “sensitive” or “resistant”, and the resulting information is stored. If conflicting information is found within a clause due to a parsing error (i.e. both sensitive and resistant terms found, or more than one antiviral specified), then the information is ignored. There are multiple cases, such as with A/Philippines/1279/2006, where more information is contained within publications on antiviral resistance that cannot currently be interpreted by the pipeline parser, and is thus ignored. In other cases, typos such as “resitant” are present, and currently not interpreted by the pipeline parser. Factoring in such information would currently require human intervention. 

### Pipeline - Geotagging 

 Location information is also sought first from the EpiFlu or FluData block in the comments section, then from the source feature block, and finally inferred from the strain name. Often, particularly with older sequences, more precise information is given in the strain name, with only country being provided in the source feature block, if present at all. Other times, more specific location information is provided within the record than within the strain name, such as with many recent samples from the USA which contain county information in addition to state. The parser makes a decision based on the number of words inside the feature block to use either the strain location identifier or the country identifier when looking up latitude and longitude. If an exact match has been found within the existing database on each of the location fields, then the query sequence is automatically tagged with the existing information; otherwise, the query is submitted to the GeoNames webservice. The top ten results are then provided to the user to choose the one that matches the current sequence. Users can also send their own query to the GeoNames webservice if none of the records is appropriate. Since only 872 of the 6857 strains initially considered had potentially distinct country and strain location fields, geotagging all 6857 sequences with this semi-automated system took approximately 2 hours, which is approximately one sequence each second, or one distinct sequence every ten seconds. This includes time for the system to query Geonames, time to manually look up some sequences in other data sources such as original publications or the Influenza Virus Resource when the place name is ambiguous, to find correct spellings when typos occur in names, and to look up additional information with services such as Google Earth when GeoNames.org could not find the place name. Google Earth was sometimes needed with sequences from locations around Perth in Western Australia, which does not appear to be well supported in the current version of GeoNames. If an ambiguous place name could not be resolved, the sequence was attributed solely to the country of origin, which is provided for almost all sequences by the NCBI Influenza Virus Resource.  Without this semi-automated parser and tagging system, it would take approximately 143 person-hours to manually geotag this dataset based on a conservative estimate of 30 seconds to manually geo-tag each record.  Keeping the system up-to-date is manageable as most sequence location fields are not distinct from previously tagged sequences and thus can be automatically geotagged.

### Pipeline – Sequence alignment and polymorphism detection 

 After parsing the full GenBank records of each sequence, amino acid alignments of homologous sequences are constructed using MUSCLE version 3.6 [10] with an increased gap opening penalty of 20 to eliminate incorrect insertion and deletion artifacts due to partial sequences. The alignments are then examined manually and cleaned of any artifacts, such as superfluous terminal gaps and non-homologous regions extending beyond the coding region. The HA alignment contains 567 amino acids and the NA alignment contains 470 amino acids.

 As we need to be able to efficiently query polymorphisms according to various query dimensions, we have constructed a simple star-schema data warehouse to contain our polymorphism information [11]. In the current version of SeqMonitor, this data warehouse has four available dimension tables for collection date, resistance, polymorphic character and location, along with a single fact table on polymorphisms. We also include the alignment position, S-OIV inclusion flag, and GI number for searching. Although often with data warehouses we do not want to keep individual identifiers such as GI, in this case the GI is useful for excluding sequences from our analysis that are exactly identical to a query sequence. In all, 3,085,598 records are present in our fact table, which is equal to every unique combination of searchable dimension values, without terminal gaps. Hierarchical searching is also supported, such as searching on region, country, or continent in the location dimension. 

### Web application – GeoLocationBLAST (GLBLAST) 

 The second module of SeqMonitor is the visualization and analysis module which allows the results of the pipeline to be explored through the web and compared with user submitted sequences. Users may either search the database for a sequence on any of the metadata fields, or they can submit their own FASTA formatted sequence to the server. On submission, BLAST is run on the sequence using the default E-value of 10 and at most the top 100 matches retrieved. Low-complexity filtering is turned off on the BLAST query due to the high similarity of the sequences. The temporal and spatial distributions of the top BLAST matches, as well as the percentage of identical amino acids are returned. The geographic distribution of BLAST results is similar to what megx.net provides for marine microbial samples (http://www,megx.net) [12]. Identical sequences are reported separately from the main BLAST results, and are excluded from further polymorphism analysis. The Google Maps API version 2 is used to provide a graphical distribution of the top BLAST matches. The year and country distribution is also provided in tabular format. 

### Web application – Polymorphism analysis

 Once the gene the query sequence is from has been identified, the user can move on to the polymorphism analysis. The query sequence is aligned to a 5-sequence reference alignment for the given gene. Again, the Google Maps API version 2 is used, this time with a custom genomic map provider, for displaying polymorphic sites along the genome. Users can specify groupings based on any metadata field in the database, such as specific antiviral resistance or inclusion in the S-OIV pandemic, in order to compare polymorphic distributions between that subset and the overall set of homologous sequences in the dataset. Unique polymorphisms in the subset are highlighted, as well as rare (defined as a character that occurs in less than half of the known sequences at that position), and polymorphic sites. Conserved positions are also easily identified by the lack of marking. From here, individual polymorphisms within each set can be selected, and the geographic and temporal distribution will be displayed. Sequences within the set can be downloaded for offline analysis. 

## Results 

### Example: Distribution of oseltamivir resistance in sequenced isolates

 Strains of the seasonal H1N1 have recently shown increased resistance to the neuraminidase inhibiter oseltamivir . Out of the 2889 NA genes stored in our data warehouse, 275 of them have been recognized as being annotated as resistant to oseltamivir, the earliest having been collected on 18 December 2006 in Georgia, USA (A/Georgia/20/2006). Of these, 6 are part of the S-OIV outbreak, out of a total 1033 S-OIV neuraminidase sequences. These strains do not appear to be sensitive to adamantane, but do not show resistance to zanamivir. The resistant isolates are localized to five separate countries (Figure 1). 

**Figure:**
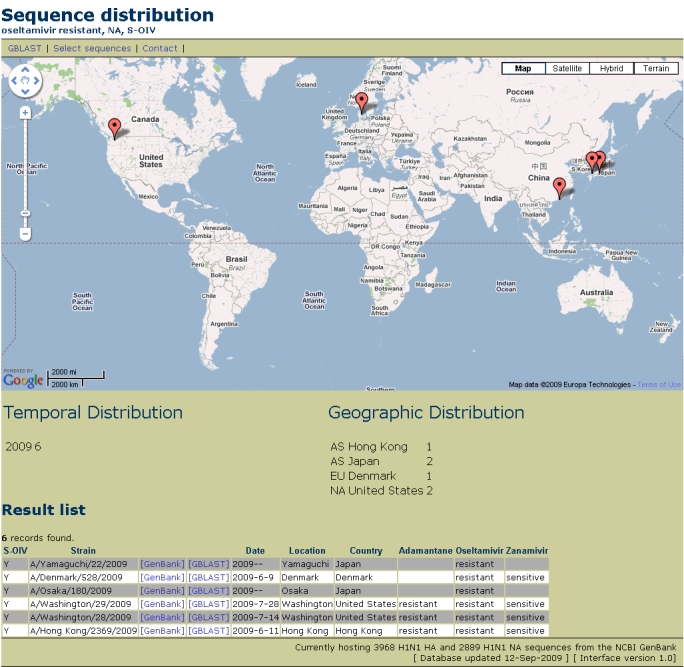
**Figure 1.** Temporal and geographic distribution of S-OIV oseltamivir-resistant neuraminidase sequences. By clicking on the Washington state marker, we can see the date of collection of each of the two sequences represented by it.

 Selecting one of the latest S-OIV sequence with oseltamivir resistance as our reference sequence, A/Washington/28/2009, we show the polymorphic site distribution against a subset of all oseltamivir resistant sequences (Figure 2). The two bars along the top of the amino acid sequence show whether the character in our query sequence is unique, a minority, a majority or conserved in either the selected subset (lower bar) or all other sequences (upper bar). Most of the minority characters in our query sequence line up in both sets, with no indication that the character is related to the metadata values selected for the set. A single site shows a rare polymorphism in the background set that is conserved in the oseltamivir resistant set, residue Y275 (Y274 in N2 numbering) (Figure 2). The presence of a tyrosine residue at this site is known to confer resistance to oseltamivir.

**Figure:**
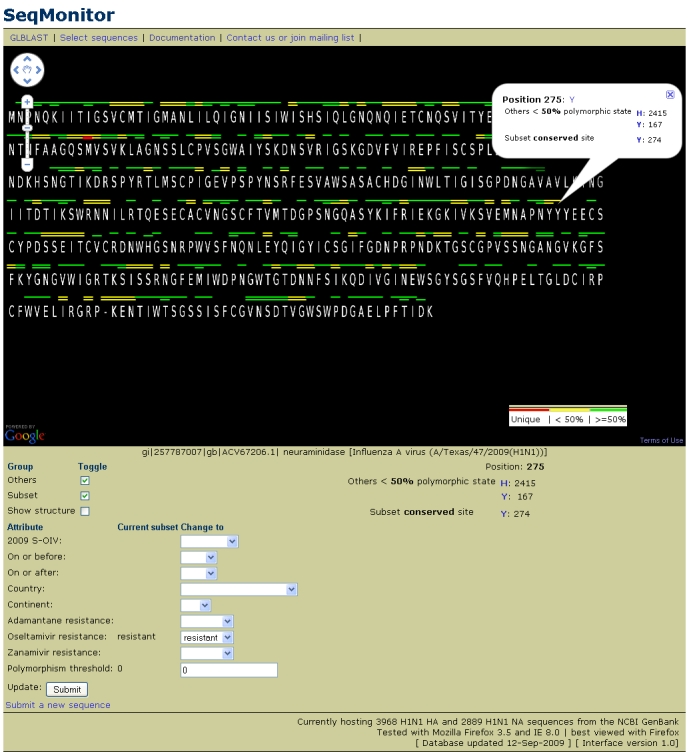
**Figure 2.**  Polymorphic sites of the amino acid sequence of A/Washington/28/2009.  The lower bar represents the subset and upper bar represents all other sequences.  Position Y275, already known to confer resistance to oseltamivir, is highlighted by a conserved subset site versus a rare polymorphism in all other sequences

 Although the most interesting residues are those conserved in one set and a minority or unique in the other, two other sites show a minority residue in the oseltamivir-resistant subset that is in the majority of all other sequences, residue R173.  This residue is present in only 5/260 (1.9%) of oseltamivir-resistant sequences, versus 1176/2339 (50.2%) of all other sequences. By clicking on the amino acid character for the 5 oseltamivir-resistant R173 sequences, we see that they are all part of the S-OIV outbreak. This indicates that the S-OIV outbreak may have the first examples of R173 in oseltamivir-resistant sequences.  By changing the subset to S-OIV, we can see that R173 is completely conserved in S-OIV sequences, and not likely related to oseltamivir resistance at all, but rather related due to coalescence. 

 Site G249 is present in 13/274 (4.7%) of oseltamivir-resistant sequences, but is present in 2089/2582 (80.9%) of all other sequences. G249 is present in all S-OIV sequences, and has been seen before in oseltamivir resistant strains in the US in 2006, and the US and Japan in 2007.  However, K249 is present in the other 261/274 (95.3%) oseltamivir-resistant sequences, but only in 440/2582 (17.0%) of other sequences.  It may be interesting to track if a G249K mutation increases fitness in S-OIV oseltamivir-resistant sequences.

### Example: Tracking the K136 residue

 Recently, NA K136 has been linked to zanamivir resistance [4].  By selecting this residue, we can view the temporal and geographic distribution of this important mutation (Figure 3).  This residue is present in 11 neuraminidase sequences available in GenBank.  None of the 11 sequences is part of the S-OIV pandemic. The earliest sequence with this mutation is from 30 May 2006, from the Philippines.  It has been present in 4 sequences in 2006, 3 in 2007 and 4 in 2008.  It has not yet been encountered in 2009.  Note that in this case, the zanamivir resistance information is contained in a linked reference, not in the GenBank record itself.  Although we can track any position's character value with SeqMonitor, the metadata are not complete if the parser cannot find the appropriate tags.  Manual curation is necessary to keep the system accurate.

**Figure:**
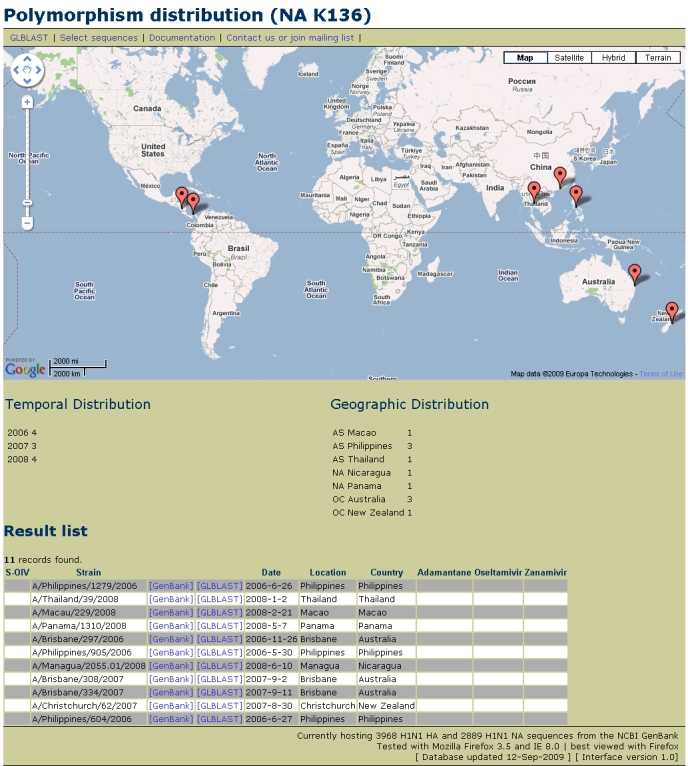
**Figure 3**.  Temporal and geographic distribution of NA K136, a mutation linked to zanamivir resistance. This mutation has been seen mainly in the southern hemisphere and tropical regions.  Interestingly, it has not been found yet in the most thoroughly sampled country, the USA, even though it could be found as early as 2006.

## Conclusion 

 The primary motivations for SeqMonitor are to facilitate the identification of characteristic mutations for a given sequence set and to detect new sequence variants as they emerge in the public databases. SeqMonitor can allow the exploitation of a growing body of general and strain-specific knowledge about the influenza A virus, as new site information can be added to the system as results are described in the literature [4] [5] [13] [14] [15] [16] [17]. Currently, the SeqMonitor web application and data warehouse supports the H1N1 hemagglutinin and neuraminidase genes, but we will be expanding the system to track other subtypes and genes, which will allow analysis of resistance to antivirals such as the M2-targeting adamantane. The system is sufficiently generic that it can be adapted to any set of homologous sequences for which annotation is available, although the clarity and reliability of the analysis are highest when sequences are very similar and differ by few or no insertion and deletion mutations.

## Funding Sources

 Development of SeqMonitor was supported in part by Genome Atlantic. NJM is supported by an NSERC postgraduate scholarship. DHP is supported by the Killam Trusts. RGB acknowledges the support of the Canada Research Chairs program. 

## Competing Interests

 The authors declare that they have no competing financial interests.  
